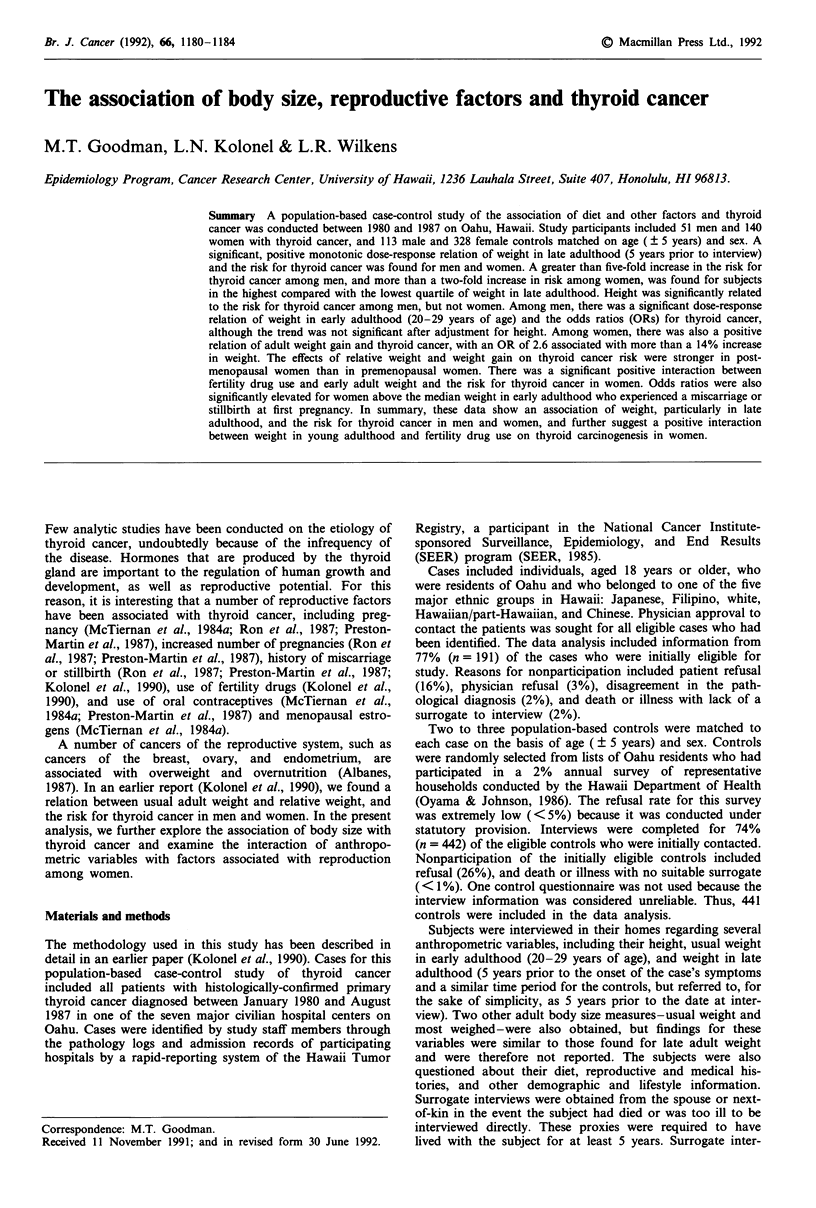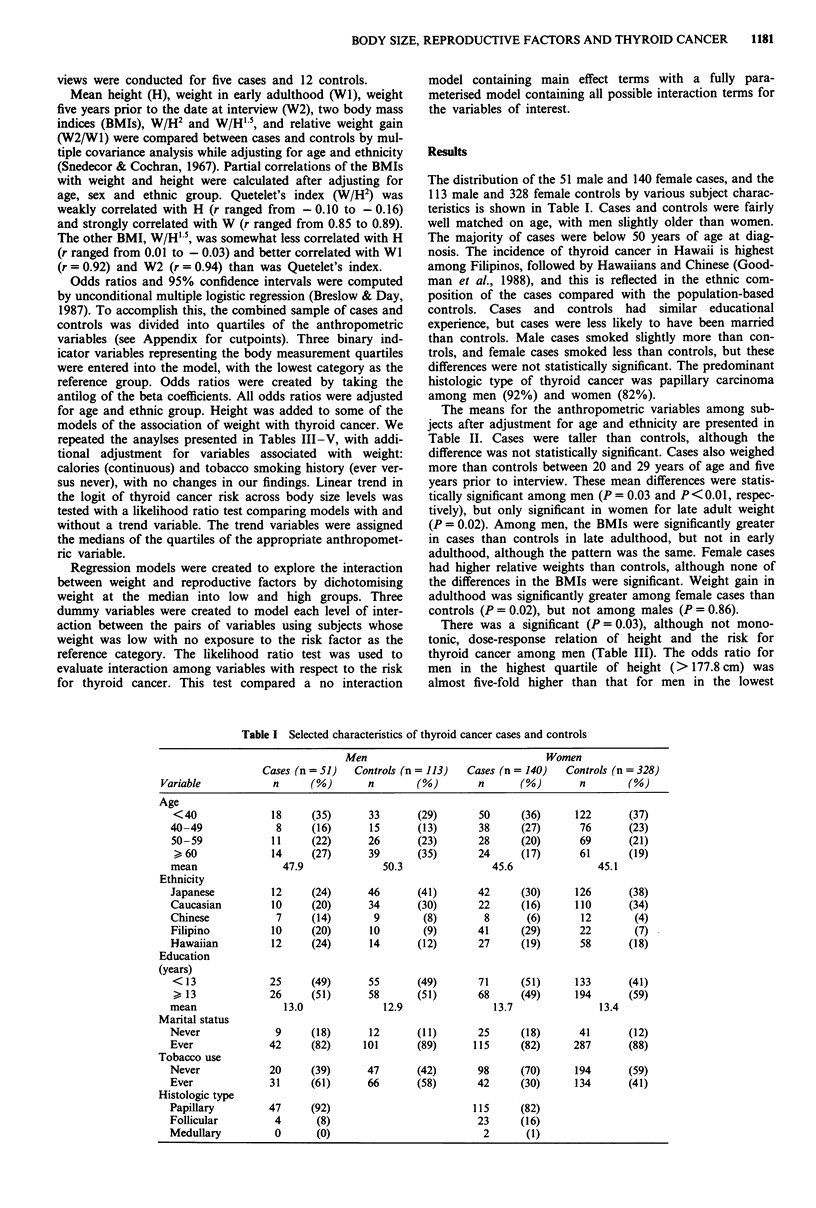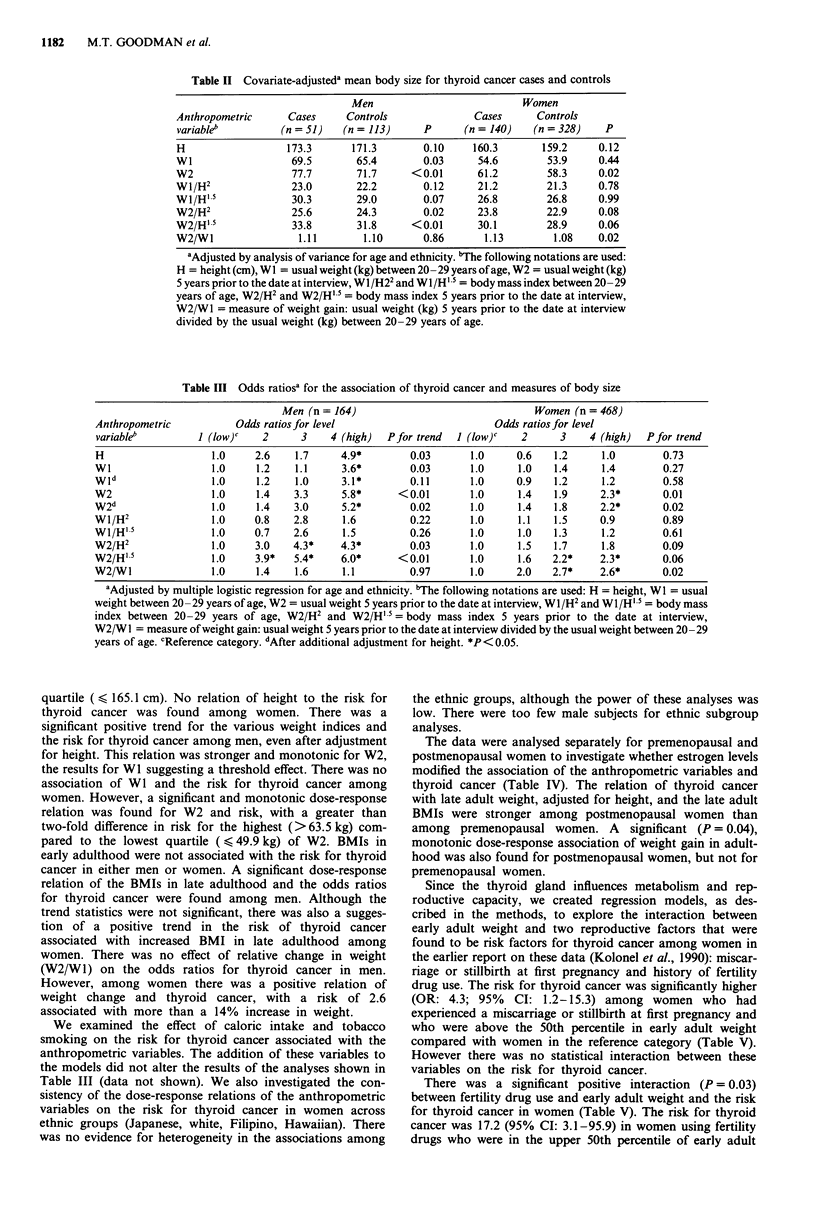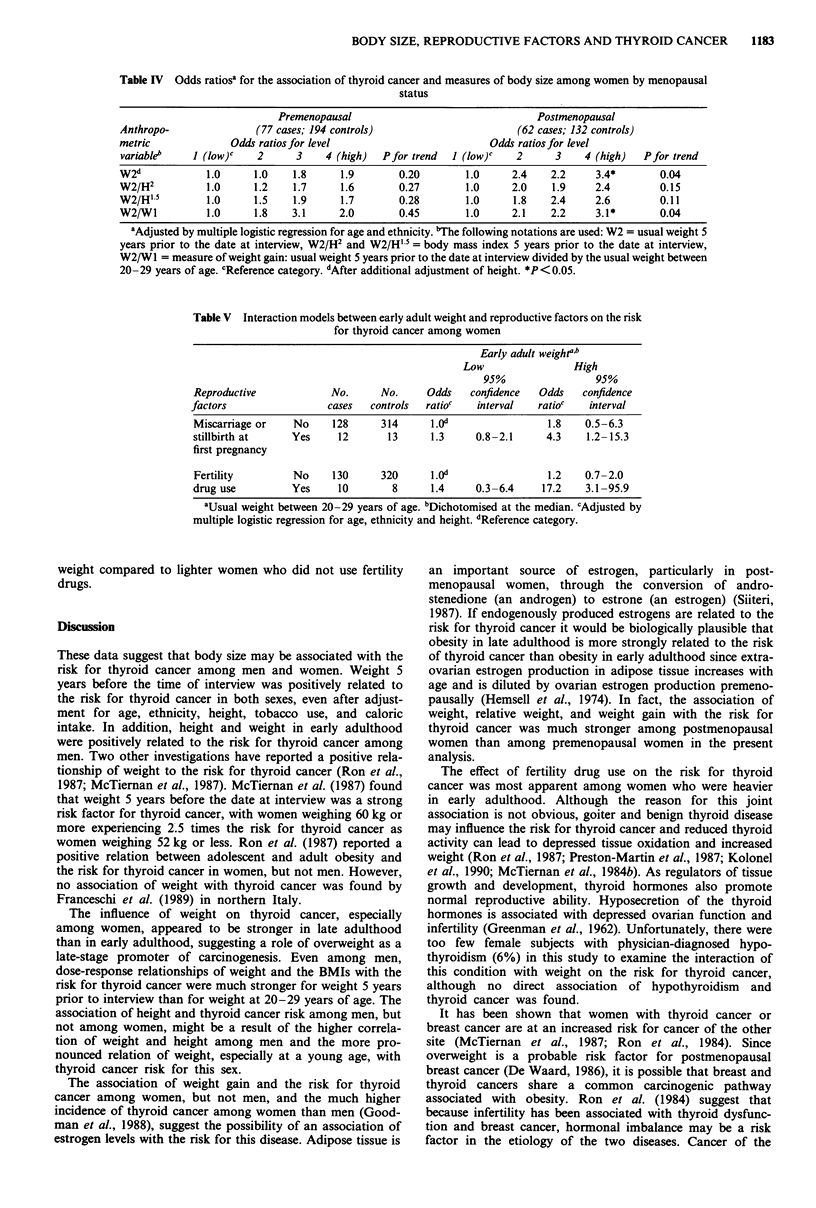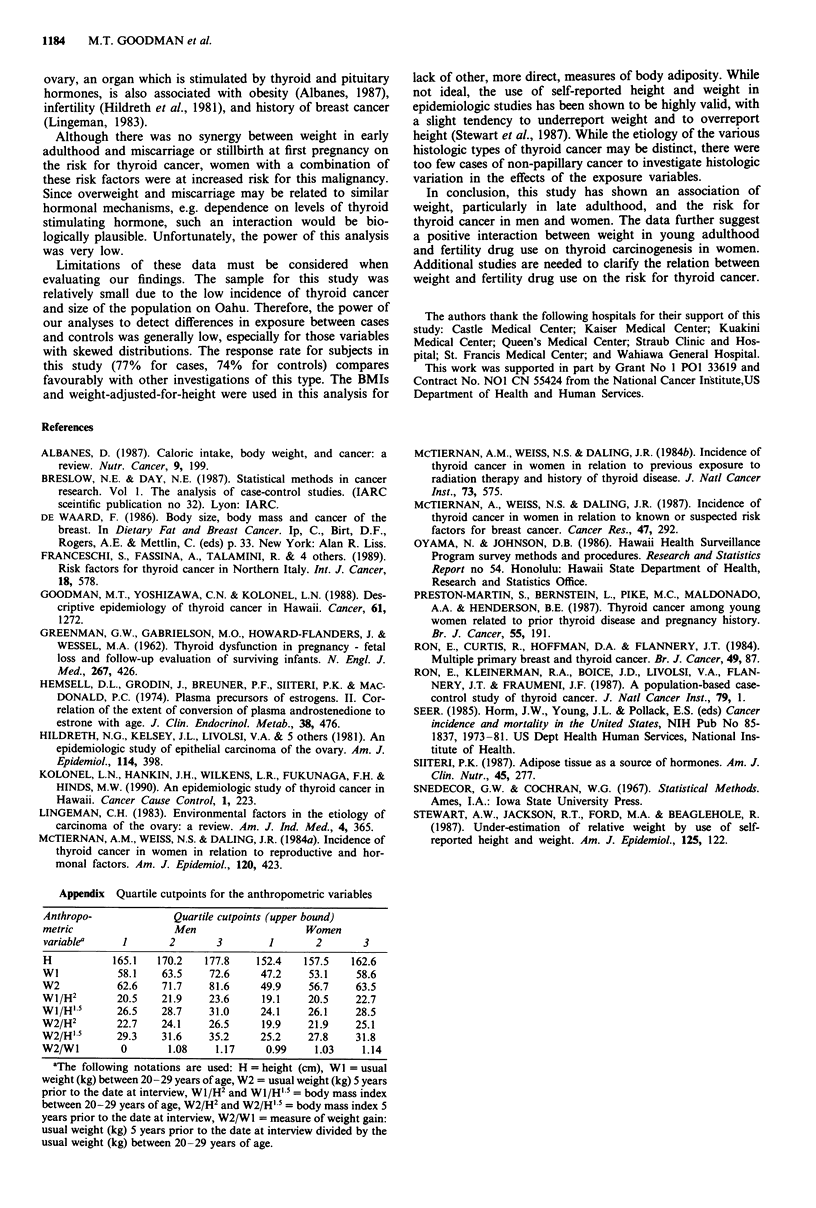# The association of body size, reproductive factors and thyroid cancer.

**DOI:** 10.1038/bjc.1992.432

**Published:** 1992-12

**Authors:** M. T. Goodman, L. N. Kolonel, L. R. Wilkens

**Affiliations:** Epidemiology Program, University of Hawaii, Honolulu 96813.

## Abstract

A population-based case-control study of the association of diet and other factors and thyroid cancer was conducted between 1980 and 1987 on Oahu, Hawaii. Study participants included 51 men and 140 women with thyroid cancer, and 113 male and 328 female controls matched on age (+/- 5 years) and sex. A significant, positive monotonic dose-response relation of weight in late adulthood (5 years prior to interview) and the risk for thyroid cancer was found for men and women. A greater than five-fold increase in the risk for thyroid cancer among men, and more than a two-fold increase in risk among women, was found for subjects in the highest compared with the lowest quartile of weight in late adulthood. Height was significantly related to the risk for thyroid cancer among men, but not women. Among men, there was a significant dose-response relation of weight in early adulthood (20-29 years of age) and the odds ratios (ORs) for thyroid cancer, although the trend was not significant after adjustment for height. Among women, there was also a positive relation of adult weight gain and thyroid cancer, with an OR of 2.6 associated with more than a 14% increase in weight. The effects of relative weight and weight gain on thyroid cancer risk were stronger in post-menopausal women than in premenopausal women. There was a significant positive interaction between fertility drug use and early adult weight and the risk for thyroid cancer in women. Odds ratios were also significantly elevated for women above the median weight in early adulthood who experienced a miscarriage or stillbirth at first pregnancy. In summary, these data show an association of weight, particularly in late adulthood, and the risk for thyroid cancer in men and women, and further suggest a positive interaction between weight in young adulthood and fertility drug use on thyroid carcinogenesis in women.


*Br. J. Cancer* (1992), **66**, 1180–1184 © Macmillan Press Ltd., 1992

### The association of body size, reproductive factors and thyroid cancer

M.T. Goodman, L.N. Kolonel & L.R. Wilkens

*Epidemiology Program, Cancer Research Center, University of Hawaii, 1236 Lauhala Street, Suite 407, Honolulu, HI 96813.*

**Summary** A population-based case-control study of the association of diet and other factors and thyroid cancer was conducted between 1980 and 1987 on Oahu, Hawaii. Study participants included 51 men and 140 women with thyroid cancer, and 113 male and 328 female controls matched on age (± 5 years) and sex. A significant, positive monotonic dose-response relation of weight in late adulthood (5 years prior to interview) and the risk for thyroid cancer was found for men and women. A greater than five-fold increase in the risk for thyroid cancer among men, and more than a two-fold increase in risk among women, was found for subjects in the highest compared with the lowest quartile of weight in late adulthood. Height was significantly related to the risk for thyroid cancer among men, but not women. Among men, there was a significant dose-response relation of weight in early adulthood (20–29 years of age) and the odds ratios (ORs) for thyroid cancer, although the trend was not significant after adjustment for height. Among women, there was also a positive relation of adult weight gain and thyroid cancer, with an OR of 2.6 associated with more than a 14% increase in weight. The effects of relative weight and weight gain on thyroid cancer risk were stronger in post-menopausal women than in premenopausal women. There was a significant positive interaction between fertility drug use and early adult weight and the risk for thyroid cancer in women. Odds ratios were also significantly elevated for women above the median weight in early adulthood who experienced a miscarriage or stillbirth at first pregnancy. In summary, these data show an association of weight, particularly in late adulthood, and the risk for thyroid cancer in men and women, and further suggest a positive interaction between weight in young adulthood and fertility drug use on thyroid carcinogenesis in women.

Few analytic studies have been conducted on the etiology of thyroid cancer, undoubtedly because of the infrequency of the disease. Hormones that are produced by the thyroid gland are important to the regulation of human growth and development, as well as reproductive potential. For this reason, it is interesting that a number of reproductive factors have been associated with thyroid cancer, including pregnancy (McTiernan *et al.*, 1984*a*; Ron *et al.*, 1987; Preston-Martin *et al.*, 1987), increased number of pregnancies (Ron *et al.*, 1987; Preston-Martin *et al.*, 1987), history of miscarriage or stillbirth (Ron *et al.*, 1987; Preston-Martin *et al.*, 1987; Kolonel *et al.*, 1990), use of fertility drugs (Kolonel *et al.*, 1990), and use of oral contraceptives (McTiernan *et al.*, 1984*a*; Preston-Martin *et al.*, 1987) and menopausal estrogens (McTiernan *et al.*, 1984*a*).

A number of cancers of the reproductive system, such as cancers of the breast, ovary, and endometrium, are associated with overweight and overnutrition (Albanes, 1987). In an earlier report (Kolonel *et al.*, 1990), we found a relation between usual adult weight and relative weight, and the risk for thyroid cancer in men and women. In the present analysis, we further explore the association of body size with thyroid cancer and examine the interaction of anthropometric variables with factors associated with reproduction among women.

### Materials and methods

The methodology used in this study has been described in detail in an earlier paper (Kolonel *et al.*, 1990). Cases for this population-based case-control study of thyroid cancer included all patients with histologically-confirmed primary thyroid cancer diagnosed between January 1980 and August 1987 in one of the seven major civilian hospital centers on Oahu. Cases were identified by study staff members through the pathology logs and admission records of participating hospitals by a rapid-reporting system of the Hawaii Tumor Registry, a participant in the National Cancer Institute-sponsored Surveillance, Epidemiology, and End Results (SEER) program (SEER, 1985).

Cases included individuals, aged 18 years or older, who were residents of Oahu and who belonged to one of the five major ethnic groups in Hawaii: Japanese, Filipino, white, Hawaiian/part-Hawaiian, and Chinese. Physician approval to contact the patients was sought for all eligible cases who had been identified. The data analysis included information from 77% ($n = 191$) of the cases who were initially eligible for study. Reasons for nonparticipation included patient refusal (16%), physician refusal (3%), disagreement in the pathological diagnosis (2%), and death or illness with lack of a surrogate to interview (2%).

Two to three population-based controls were matched to each case on the basis of age (± 5 years) and sex. Controls were randomly selected from lists of Oahu residents who had participated in a 2% annual survey of representative households conducted by the Hawaii Department of Health (Oyama & Johnson, 1986). The refusal rate for this survey was extremely low (<5%) because it was conducted under statutory provision. Interviews were completed for 74% ($n = 442$) of the eligible controls who were initially contacted. Nonparticipation of the initially eligible controls included refusal (26%), and death or illness with no suitable surrogate (<1%). One control questionnaire was not used because the interview information was considered unreliable. Thus, 441 controls were included in the data analysis.

Subjects were interviewed in their homes regarding several anthropometric variables, including their height, usual weight in early adulthood (20–29 years of age), and weight in late adulthood (5 years prior to the onset of the case's symptoms and a similar time period for the controls, but referred to, for the sake of simplicity, as 5 years prior to the date at interview). Two other adult body size measures–usual weight and most weighed–were also obtained, but findings for these variables were similar to those found for late adult weight and were therefore not reported. The subjects were also questioned about their diet, reproductive and medical histories, and other demographic and lifestyle information. Surrogate interviews were obtained from the spouse or next-of-kin in the event the subject had died or was too ill to be interviewed directly. These proxies were required to have lived with the subject for at least 5 years. Surrogate inter-

Correspondence: M.T. Goodman.
Received 11 November 1991; and in revised form 30 June 1992.

views were conducted for five cases and 12 controls.

Mean height (H), weight in early adulthood (W1), weight five years prior to the date at interview (W2), two body mass indices (BMIs), $W/H^2$ and $W/H^{1.5}$, and relative weight gain (W2/W1) were compared between cases and controls by multiple covariance analysis while adjusting for age and ethnicity (Snedecor & Cochran, 1967). Partial correlations of the BMIs with weight and height were calculated after adjusting for age, sex and ethnic group. Quetelet's index ($W/H^2$) was weakly correlated with H ($r$ ranged from $-0.10$ to $-0.16$) and strongly correlated with W ($r$ ranged from 0.85 to 0.89). The other BMI, $W/H^{1.5}$, was somewhat less correlated with H ($r$ ranged from 0.01 to $-0.03$) and better correlated with W1 ($r = 0.92$) and W2 ($r = 0.94$) than was Quetelet's index.

Odds ratios and 95% confidence intervals were computed by unconditional multiple logistic regression (Breslow & Day, 1987). To accomplish this, the combined sample of cases and controls was divided into quartiles of the anthropometric variables (see Appendix for cutpoints). Three binary indicator variables representing the body measurement quartiles were entered into the model, with the lowest category as the reference group. Odds ratios were created by taking the antilog of the beta coefficients. All odds ratios were adjusted for age and ethnic group. Height was added to some of the models of the association of weight with thyroid cancer. We repeated the anaylses presented in Tables III–V, with additional adjustment for variables associated with weight: calories (continuous) and tobacco smoking history (ever versus never), with no changes in our findings. Linear trend in the logit of thyroid cancer risk across body size levels was tested with a likelihood ratio test comparing models with and without a trend variable. The trend variables were assigned the medians of the quartiles of the appropriate anthropometric variable.

Regression models were created to explore the interaction between weight and reproductive factors by dichotomising weight at the median into low and high groups. Three dummy variables were created to model each level of interaction between the pairs of variables using subjects whose weight was low with no exposure to the risk factor as the reference category. The likelihood ratio test was used to evaluate interaction among variables with respect to the risk for thyroid cancer. This test compared a no interaction model containing main effect terms with a fully parameterised model containing all possible interaction terms for the variables of interest.

### Results

The distribution of the 51 male and 140 female cases, and the 113 male and 328 female controls by various subject characteristics is shown in Table I. Cases and controls were fairly well matched on age, with men slightly older than women. The majority of cases were below 50 years of age at diagnosis. The incidence of thyroid cancer in Hawaii is highest among Filipinos, followed by Hawaiians and Chinese (Goodman *et al.*, 1988), and this is reflected in the ethnic composition of the cases compared with the population-based controls. Cases and controls had similar educational experience, but cases were less likely to have been married than controls. Male cases smoked slightly more than controls, and female cases smoked less than controls, but these differences were not statistically significant. The predominant histologic type of thyroid cancer was papillary carcinoma among men (92%) and women (82%).

The means for the anthropometric variables among subjects after adjustment for age and ethnicity are presented in Table II. Cases were taller than controls, although the difference was not statistically significant. Cases also weighed more than controls between 20 and 29 years of age and five years prior to interview. These mean differences were statistically significant among men ($P = 0.03$ and $P < 0.01$, respectively), but only significant in women for late adult weight ($P = 0.02$). Among men, the BMIs were significantly greater in cases than controls in late adulthood, but not in early adulthood, although the pattern was the same. Female cases had higher relative weights than controls, although none of the differences in the BMIs were significant. Weight gain in adulthood was significantly greater among female cases than controls ($P = 0.02$), but not among males ($P = 0.86$).

There was a significant ($P = 0.03$), although not monotonic, dose-response relation of height and the risk for thyroid cancer among men (Table III). The odds ratio for men in the highest quartile of height (>177.8 cm) was almost five-fold higher than that for men in the lowest

**Table I** Selected characteristics of thyroid cancer cases and controls

| | *Men* | | | | *Women* | | | |
|---|---|---|---|---|---|---|---|---|
| | *Cases* (n = *51*) | | *Controls* (n = *113*) | | *Cases* (n = *140*) | | *Controls* (n = *328*) | |
| *Variable* | *n* | *(%)* | *n* | *(%)* | *n* | *(%)* | *n* | *(%)* |
| Age | | | | | | | | |
| <40 | 18 | (35) | 33 | (29) | 50 | (36) | 122 | (37) |
| 40–49 | 8 | (16) | 15 | (13) | 38 | (27) | 76 | (23) |
| 50–59 | 11 | (22) | 26 | (23) | 28 | (20) | 69 | (21) |
| ⩾60 | 14 | (27) | 39 | (35) | 24 | (17) | 61 | (19) |
| mean | 47.9 | | 50.3 | | 45.6 | | 45.1 | |
| Ethnicity | | | | | | | | |
| Japanese | 12 | (24) | 46 | (41) | 42 | (30) | 126 | (38) |
| Caucasian | 10 | (20) | 34 | (30) | 22 | (16) | 110 | (34) |
| Chinese | 7 | (14) | 9 | (8) | 8 | (6) | 12 | (4) |
| Filipino | 10 | (20) | 10 | (9) | 41 | (29) | 22 | (7) |
| Hawaiian | 12 | (24) | 14 | (12) | 27 | (19) | 58 | (18) |
| Education (years) | | | | | | | | |
| <13 | 25 | (49) | 55 | (49) | 71 | (51) | 133 | (41) |
| ⩾13 | 26 | (51) | 58 | (51) | 68 | (49) | 194 | (59) |
| mean | 13.0 | | 12.9 | | 13.7 | | 13.4 | |
| Marital status | | | | | | | | |
| Never | 9 | (18) | 12 | (11) | 25 | (18) | 41 | (12) |
| Ever | 42 | (82) | 101 | (89) | 115 | (82) | 287 | (88) |
| Tobacco use | | | | | | | | |
| Never | 20 | (39) | 47 | (42) | 98 | (70) | 194 | (59) |
| Ever | 31 | (61) | 66 | (58) | 42 | (30) | 134 | (41) |
| Histologic type | | | | | | | | |
| Papillary | 47 | (92) | | | 115 | (82) | | |
| Follicular | 4 | (8) | | | 23 | (16) | | |
| Medullary | 0 | (0) | | | 2 | (1) | | |

**Table II** Covariate-adjusted[a] mean body size for thyroid cancer cases and controls

| Anthropometric variable[b] | Men Cases (n = 51) | Men Controls (n = 113) | P | Women Cases (n = 140) | Women Controls (n = 328) | P |
|---|---|---|---|---|---|---|
| H | 173.3 | 171.3 | 0.10 | 160.3 | 159.2 | 0.12 |
| W1 | 69.5 | 65.4 | 0.03 | 54.6 | 53.9 | 0.44 |
| W2 | 77.7 | 71.7 | <0.01 | 61.2 | 58.3 | 0.02 |
| $W1/H^2$ | 23.0 | 22.2 | 0.12 | 21.2 | 21.3 | 0.78 |
| $W1/H^{1.5}$ | 30.3 | 29.0 | 0.07 | 26.8 | 26.8 | 0.99 |
| $W2/H^2$ | 25.6 | 24.3 | 0.02 | 23.8 | 22.9 | 0.08 |
| $W2/H^{1.5}$ | 33.8 | 31.8 | <0.01 | 30.1 | 28.9 | 0.06 |
| W2/W1 | 1.11 | 1.10 | 0.86 | 1.13 | 1.08 | 0.02 |

[a]Adjusted by analysis of variance for age and ethnicity. [b]The following notations are used: H = height (cm), W1 = usual weight (kg) between 20–29 years of age, W2 = usual weight (kg) 5 years prior to the date at interview, $W1/H2^2$ and $W1/H^{1.5}$ = body mass index between 20–29 years of age, $W2/H^2$ and $W2/H^{1.5}$ = body mass index 5 years prior to the date at interview, W2/W1 = measure of weight gain: usual weight (kg) 5 years prior to the date at interview divided by the usual weight (kg) between 20–29 years of age.

**Table III** Odds ratios[a] for the association of thyroid cancer and measures of body size

| Anthropometric variable[b] | Men (n = 164) Odds ratios for level 1 (low)[c] | 2 | 3 | 4 (high) | P for trend | Women (n = 468) Odds ratios for level 1 (low)[c] | 2 | 3 | 4 (high) | P for trend |
|---|---|---|---|---|---|---|---|---|---|---|
| H | 1.0 | 2.6 | 1.7 | 4.9* | 0.03 | 1.0 | 0.6 | 1.2 | 1.0 | 0.73 |
| W1 | 1.0 | 1.2 | 1.1 | 3.6* | 0.03 | 1.0 | 1.0 | 1.4 | 1.4 | 0.27 |
| W1[d] | 1.0 | 1.2 | 1.0 | 3.1* | 0.11 | 1.0 | 0.9 | 1.2 | 1.2 | 0.58 |
| W2 | 1.0 | 1.4 | 3.3 | 5.8* | <0.01 | 1.0 | 1.4 | 1.9 | 2.3* | 0.01 |
| W2[d] | 1.0 | 1.4 | 3.0 | 5.2* | 0.02 | 1.0 | 1.4 | 1.8 | 2.2* | 0.02 |
| $W1/H^2$ | 1.0 | 0.8 | 2.8 | 1.6 | 0.22 | 1.0 | 1.1 | 1.5 | 0.9 | 0.89 |
| $W1/H^{1.5}$ | 1.0 | 0.7 | 2.6 | 1.5 | 0.26 | 1.0 | 1.0 | 1.3 | 1.2 | 0.61 |
| $W2/H^2$ | 1.0 | 3.0 | 4.3* | 4.3* | 0.03 | 1.0 | 1.5 | 1.7 | 1.8 | 0.09 |
| $W2/H^{1.5}$ | 1.0 | 3.9* | 5.4* | 6.0* | <0.01 | 1.0 | 1.6 | 2.2* | 2.3* | 0.06 |
| W2/W1 | 1.0 | 1.4 | 1.6 | 1.1 | 0.97 | 1.0 | 2.0 | 2.7* | 2.6* | 0.02 |

[a]Adjusted by multiple logistic regression for age and ethnicity. [b]The following notations are used: H = height, W1 = usual weight between 20–29 years of age, W2 = usual weight 5 years prior to the date at interview, $W1/H^2$ and $W1/H^{1.5}$ = body mass index between 20–29 years of age, $W2/H^2$ and $W2/H^{1.5}$ = body mass index 5 years prior to the date at interview, W2/W1 = measure of weight gain: usual weight 5 years prior to the date at interview divided by the usual weight between 20–29 years of age. [c]Reference category. [d]After additional adjustment for height. *$P < 0.05$.

quartile ($\leqslant$ 165.1 cm). No relation of height to the risk for thyroid cancer was found among women. There was a significant positive trend for the various weight indices and the risk for thyroid cancer among men, even after adjustment for height. This relation was stronger and monotonic for W2, the results for W1 suggesting a threshold effect. There was no association of W1 and the risk for thyroid cancer among women. However, a significant and monotonic dose-response relation was found for W2 and risk, with a greater than two-fold difference in risk for the highest (>63.5 kg) compared to the lowest quartile ($\leqslant$49.9 kg) of W2. BMIs in early adulthood were not associated with the risk for thyroid cancer in either men or women. A significant dose-response relation of the BMIs in late adulthood and the odds ratios for thyroid cancer were found among men. Although the trend statistics were not significant, there was also a suggestion of a positive trend in the risk of thyroid cancer associated with increased BMI in late adulthood among women. There was no effect of relative change in weight (W2/W1) on the odds ratios for thyroid cancer in men. However, among women there was a positive relation of weight change and thyroid cancer, with a risk of 2.6 associated with more than a 14% increase in weight.

We examined the effect of caloric intake and tobacco smoking on the risk for thyroid cancer associated with the anthropometric variables. The addition of these variables to the models did not alter the results of the analyses shown in Table III (data not shown). We also investigated the consistency of the dose-response relations of the anthropometric variables on the risk for thyroid cancer in women across ethnic groups (Japanese, white, Filipino, Hawaiian). There was no evidence for heterogeneity in the associations among the ethnic groups, although the power of these analyses was low. There were too few male subjects for ethnic subgroup analyses.

The data were analysed separately for premenopausal and postmenopausal women to investigate whether estrogen levels modified the association of the anthropometric variables and thyroid cancer (Table IV). The relation of thyroid cancer with late adult weight, adjusted for height, and the late adult BMIs were stronger among postmenopausal women than among premenopausal women. A significant ($P = 0.04$), monotonic dose-response association of weight gain in adulthood was also found for postmenopausal women, but not for premenopausal women.

Since the thyroid gland influences metabolism and reproductive capacity, we created regression models, as described in the methods, to explore the interaction between early adult weight and two reproductive factors that were found to be risk factors for thyroid cancer among women in the earlier report on these data (Kolonel *et al.*, 1990): miscarriage or stillbirth at first pregnancy and history of fertility drug use. The risk for thyroid cancer was significantly higher (OR: 4.3; 95% CI: 1.2–15.3) among women who had experienced a miscarriage or stillbirth at first pregnancy and who were above the 50th percentile in early adult weight compared with women in the reference category (Table V). However there was no statistical interaction between these variables on the risk for thyroid cancer.

There was a significant positive interaction ($P = 0.03$) between fertility drug use and early adult weight and the risk for thyroid cancer in women (Table V). The risk for thyroid cancer was 17.2 (95% CI: 3.1–95.9) in women using fertility drugs who were in the upper 50th percentile of early adult

**Table IV** Odds ratios[a] for the association of thyroid cancer and measures of body size among women by menopausal status

| Anthropometric variable[b] | Premenopausal (77 cases; 194 controls) Odds ratios for level 1 (low)[c] | 2 | 3 | 4 (high) | P for trend | Postmenopausal (62 cases; 132 controls) Odds ratios for level 1 (low)[c] | 2 | 3 | 4 (high) | P for trend |
|---|---|---|---|---|---|---|---|---|---|---|
| W2[d] | 1.0 | 1.0 | 1.8 | 1.9 | 0.20 | 1.0 | 2.4 | 2.2 | 3.4* | 0.04 |
| $W2/H^2$ | 1.0 | 1.2 | 1.7 | 1.6 | 0.27 | 1.0 | 2.0 | 1.9 | 2.4 | 0.15 |
| $W2/H^{1.5}$ | 1.0 | 1.5 | 1.9 | 1.7 | 0.28 | 1.0 | 1.8 | 2.4 | 2.6 | 0.11 |
| W2/W1 | 1.0 | 1.8 | 3.1 | 2.0 | 0.45 | 1.0 | 2.1 | 2.2 | 3.1* | 0.04 |

[a]Adjusted by multiple logistic regression for age and ethnicity. [b]The following notations are used: W2 = usual weight 5 years prior to the date at interview, $W2/H^2$ and $W2/H^{1.5}$ = body mass index 5 years prior to the date at interview, W2/W1 = measure of weight gain: usual weight 5 years prior to the date at interview divided by the usual weight between 20–29 years of age. [c]Reference category. [d]After additional adjustment of height. $^*P<0.05$.

**Table V** Interaction models between early adult weight and reproductive factors on the risk for thyroid cancer among women

| Reproductive factors | | No. cases | No. controls | Early adult weight[a,b] Low: Odds ratio[c] | Low: 95% confidence interval | High: Odds ratio[c] | High: 95% confidence interval |
|---|---|---|---|---|---|---|---|
| Miscarriage or | No | 128 | 314 | 1.0[d] | | 1.8 | 0.5–6.3 |
| stillbirth at first pregnancy | Yes | 12 | 13 | 1.3 | 0.8–2.1 | 4.3 | 1.2–15.3 |
| Fertility | No | 130 | 320 | 1.0[d] | | 1.2 | 0.7–2.0 |
| drug use | Yes | 10 | 8 | 1.4 | 0.3–6.4 | 17.2 | 3.1–95.9 |

[a]Usual weight between 20–29 years of age. [b]Dichotomised at the median. [c]Adjusted by multiple logistic regression for age, ethnicity and height. [d]Reference category.

weight compared to lighter women who did not use fertility drugs.

### Discussion

These data suggest that body size may be associated with the risk for thyroid cancer among men and women. Weight 5 years before the time of interview was positively related to the risk for thyroid cancer in both sexes, even after adjustment for age, ethnicity, height, tobacco use, and caloric intake. In addition, height and weight in early adulthood were positively related to the risk for thyroid cancer among men. Two other investigations have reported a positive relationship of weight to the risk for thyroid cancer (Ron *et al.*, 1987; McTiernan *et al.*, 1987). McTiernan *et al.* (1987) found that weight 5 years before the date at interview was a strong risk factor for thyroid cancer, with women weighing 60 kg or more experiencing 2.5 times the risk for thyroid cancer as women weighing 52 kg or less. Ron *et al.* (1987) reported a positive relation between adolescent and adult obesity and the risk for thyroid cancer in women, but not men. However, no association of weight with thyroid cancer was found by Franceschi *et al.* (1989) in northern Italy.

The influence of weight on thyroid cancer, especially among women, appeared to be stronger in late adulthood than in early adulthood, suggesting a role of overweight as a late-stage promoter of carcinogenesis. Even among men, dose-response relationships of weight and the BMIs with the risk for thyroid cancer were much stronger for weight 5 years prior to interview than for weight at 20–29 years of age. The association of height and thyroid cancer risk among men, but not among women, might be a result of the higher correlation of weight and height among men and the more pronounced relation of weight, especially at a young age, with thyroid cancer risk for this sex.

The association of weight gain and the risk for thyroid cancer among women, but not men, and the much higher incidence of thyroid cancer among women than men (Goodman *et al.*, 1988), suggest the possibility of an association of estrogen levels with the risk for this disease. Adipose tissue is an important source of estrogen, particularly in postmenopausal women, through the conversion of androstenedione (an androgen) to estrone (an estrogen) (Siiteri, 1987). If endogenously produced estrogens are related to the risk for thyroid cancer it would be biologically plausible that obesity in late adulthood is more strongly related to the risk of thyroid cancer than obesity in early adulthood since extra-ovarian estrogen production in adipose tissue increases with age and is diluted by ovarian estrogen production premenopausally (Hemsell *et al.*, 1974). In fact, the association of weight, relative weight, and weight gain with the risk for thyroid cancer was much stronger among postmenopausal women than among premenopausal women in the present analysis.

The effect of fertility drug use on the risk for thyroid cancer was most apparent among women who were heavier in early adulthood. Although the reason for this joint association is not obvious, goiter and benign thyroid disease may influence the risk for thyroid cancer and reduced thyroid activity can lead to depressed tissue oxidation and increased weight (Ron *et al.*, 1987; Preston-Martin *et al.*, 1987; Kolonel *et al.*, 1990; McTiernan *et al.*, 1984*b*). As regulators of tissue growth and development, thyroid hormones also promote normal reproductive ability. Hyposecretion of the thyroid hormones is associated with depressed ovarian function and infertility (Greenman *et al.*, 1962). Unfortunately, there were too few female subjects with physician-diagnosed hypothyroidism (6%) in this study to examine the interaction of this condition with weight on the risk for thyroid cancer, although no direct association of hypothyroidism and thyroid cancer was found.

It has been shown that women with thyroid cancer or breast cancer are at an increased risk for cancer of the other site (McTiernan *et al.*, 1987; Ron *et al.*, 1984). Since overweight is a probable risk factor for postmenopausal breast cancer (De Waard, 1986), it is possible that breast and thyroid cancers share a common carcinogenic pathway associated with obesity. Ron *et al.* (1984) suggest that because infertility has been associated with thyroid dysfunction and breast cancer, hormonal imbalance may be a risk factor in the etiology of the two diseases. Cancer of the ovary, an organ which is stimulated by thyroid and pituitary hormones, is also associated with obesity (Albanes, 1987), infertility (Hildreth *et al.*, 1981), and history of breast cancer (Lingeman, 1983).

Although there was no synergy between weight in early adulthood and miscarriage or stillbirth at first pregnancy on the risk for thyroid cancer, women with a combination of these risk factors were at increased risk for this malignancy. Since overweight and miscarriage may be related to similar hormonal mechanisms, e.g. dependence on levels of thyroid stimulating hormone, such an interaction would be biologically plausible. Unfortunately, the power of this analysis was very low.

Limitations of these data must be considered when evaluating our findings. The sample for this study was relatively small due to the low incidence of thyroid cancer and size of the population on Oahu. Therefore, the power of our analyses to detect differences in exposure between cases and controls was generally low, especially for those variables with skewed distributions. The response rate for subjects in this study (77% for cases, 74% for controls) compares favourably with other investigations of this type. The BMIs and weight-adjusted-for-height were used in this analysis for lack of other, more direct, measures of body adiposity. While not ideal, the use of self-reported height and weight in epidemiologic studies has been shown to be highly valid, with a slight tendency to underreport weight and to overreport height (Stewart *et al.*, 1987). While the etiology of the various histologic types of thyroid cancer may be distinct, there were too few cases of non-papillary cancer to investigate histologic variation in the effects of the exposure variables.

In conclusion, this study has shown an association of weight, particularly in late adulthood, and the risk for thyroid cancer in men and women. The data further suggest a positive interaction between weight in young adulthood and fertility drug use on thyroid carcinogenesis in women. Additional studies are needed to clarify the relation between weight and fertility drug use on the risk for thyroid cancer.

The authors thank the following hospitals for their support of this study: Castle Medical Center; Kaiser Medical Center; Kuakini Medical Center; Queen's Medical Center; Straub Clinic and Hospital; St. Francis Medical Center; and Wahiawa General Hospital.

This work was supported in part by Grant No 1 PO1 33619 and Contract No. NO1 CN 55424 from the National Cancer Institute,US Department of Health and Human Services.

### References

ALBANES, D. (1987). Caloric intake, body weight, and cancer: a review. *Nutr. Cancer*, **9**, 199.

BRESLOW, N.E. & DAY, N.E. (1987). Statistical methods in cancer research. Vol 1. The analysis of case-control studies. (IARC sceintific publication no 32). Lyon: IARC.

DE WAARD, F. (1986). Body size, body mass and cancer of the breast. In *Dietary Fat and Breast Cancer*. Ip, C., Birt, D.F., Rogers, A.E. & Mettlin, C. (eds) p. 33. New York: Alan R. Liss.

FRANCESCHI, S., FASSINA, A., TALAMINI, R. & 4 others. (1989). Risk factors for thyroid cancer in Northern Italy. *Int. J. Cancer*, **18**, 578.

GOODMAN, M.T., YOSHIZAWA, C.N. & KOLONEL, L.N. (1988). Descriptive epidemiology of thyroid cancer in Hawaii. *Cancer*, **61**, 1272.

GREENMAN, G.W., GABRIELSON, M.O., HOWARD-FLANDERS, J. & WESSEL, M.A. (1962). Thyroid dysfunction in pregnancy - fetal loss and follow-up evaluation of surviving infants. *N. Engl. J. Med.*, **267**, 426.

HEMSELL, D.L., GRODIN, J., BREUNER, P.F., SIITERI, P.K. & MACDONALD, P.C. (1974). Plasma precursors of estrogens. II. Correlation of the extent of conversion of plasma androstenedione to estrone with age. *J. Clin. Endocrinol. Metab.*, **38**, 476.

HILDRETH, N.G., KELSEY, J.L., LIVOLSI, V.A. & 5 others (1981). An epidemiologic study of epithelial carcinoma of the ovary. *Am. J. Epidemiol.*, **114**, 398.

KOLONEL, L.N., HANKIN, J.H., WILKENS, L.R., FUKUNAGA, F.H. & HINDS, M.W. (1990). An epidemiologic study of thyroid cancer in Hawaii. *Cancer Cause Control*, **1**, 223.

LINGEMAN, C.H. (1983). Environmental factors in the etiology of carcinoma of the ovary: a review. *Am. J. Ind. Med.*, **4**, 365.

MCTIERNAN, A.M., WEISS, N.S. & DALING, J.R. (1984*a*). Incidence of thyroid cancer in women in relation to reproductive and hormonal factors. *Am. J. Epidemiol.*, **120**, 423.

MCTIERNAN, A.M., WEISS, N.S. & DALING, J.R. (1984*b*). Incidence of thyroid cancer in women in relation to previous exposure to radiation therapy and history of thyroid disease. *J. Natl Cancer Inst.*, **73**, 575.

MCTIERNAN, A., WEISS, N.S. & DALING, J.R. (1987). Incidence of thyroid cancer in women in relation to known or suspected risk factors for breast cancer. *Cancer Res.*, **47**, 292.

OYAMA, N. & JOHNSON, D.B. (1986). Hawaii Health Surveillance Program survey methods and procedures. *Research and Statistics Report* no 54. Honolulu: Hawaii State Department of Health, Research and Statistics Office.

PRESTON-MARTIN, S., BERNSTEIN, L., PIKE, M.C., MALDONADO, A.A. & HENDERSON, B.E. (1987). Thyroid cancer among young women related to prior thyroid disease and pregnancy history. *Br. J. Cancer*, **55**, 191.

RON, E., CURTIS, R., HOFFMAN, D.A. & FLANNERY, J.T. (1984). Multiple primary breast and thyroid cancer. *Br. J. Cancer*, **49**, 87.

RON, E., KLEINERMAN, R.A., BOICE, J.D., LIVOLSI, V.A., FLANNERY, J.T. & FRAUMENI, J.F. (1987). A population-based case-control study of thyroid cancer. *J. Natl Cancer Inst.*, **79**, 1.

SEER. (1985). Horm, J.W., Young, J.L. & Pollack, E.S. (eds) *Cancer incidence and mortality in the United States*, NIH Pub No 85-1837, 1973-81. US Dept Health Human Services, National Institute of Health.

SIITERI, P.K. (1987). Adipose tissue as a source of hormones. *Am. J. Clin. Nutr.*, **45**, 277.

SNEDECOR, G.W. & COCHRAN, W.G. (1967). *Statistical Methods*. Ames, I.A.: Iowa State University Press.

STEWART, A.W., JACKSON, R.T., FORD, M.A. & BEAGLEHOLE, R. (1987). Under-estimation of relative weight by use of self-reported height and weight. *Am. J. Epidemiol.*, **125**, 122.

**Appendix** Quartile cutpoints for the anthropometric variables

| *Anthropometric variable*[a] | *Quartile cutpoints (upper bound)* Men 1 | 2 | 3 | Women 1 | 2 | 3 |
|---|---|---|---|---|---|---|
| H | 165.1 | 170.2 | 177.8 | 152.4 | 157.5 | 162.6 |
| W1 | 58.1 | 63.5 | 72.6 | 47.2 | 53.1 | 58.6 |
| W2 | 62.6 | 71.7 | 81.6 | 49.9 | 56.7 | 63.5 |
| $W1/H^2$ | 20.5 | 21.9 | 23.6 | 19.1 | 20.5 | 22.7 |
| $W1/H^{1.5}$ | 26.5 | 28.7 | 31.0 | 24.1 | 26.1 | 28.5 |
| $W2/H^2$ | 22.7 | 24.1 | 26.5 | 19.9 | 21.9 | 25.1 |
| $W2/H^{1.5}$ | 29.3 | 31.6 | 35.2 | 25.2 | 27.8 | 31.8 |
| W2/W1 | 0 | 1.08 | 1.17 | 0.99 | 1.03 | 1.14 |

[a]The following notations are used: H = height (cm), W1 = usual weight (kg) between 20–29 years of age, W2 = usual weight (kg) 5 years prior to the date at interview, $W1/H^2$ and $W1/H^{1.5}$ = body mass index between 20–29 years of age, $W2/H^2$ and $W2/H^{1.5}$ = body mass index 5 years prior to the date at interview, W2/W1 = measure of weight gain: usual weight (kg) 5 years prior to the date at interview divided by the usual weight (kg) between 20–29 years of age.